# Mild gestational diabetes in pregnancy and the adipoinsular axis in babies born to mothers in the ACHOIS randomised controlled trial

**DOI:** 10.1186/1471-2431-7-18

**Published:** 2007-04-12

**Authors:** Louise K Pirc, Julie A Owens, Caroline A Crowther, Kristyn Willson, Miles J De Blasio, Jeffrey S Robinson

**Affiliations:** 1 Discipline of Obstetrics and Gynaecology, The University of Adelaide, Women's and Children's Hospital, King William Road, North Adelaide, South Australia, SA 5005, Australia

## Abstract

**Background:**

Mild gestational diabetes is a common complication of pregnancy, affecting up to 9% of pregnant women. Treatment of mild GDM is known to reduce adverse perinatal outcomes such as macrosomia and associated birth injuries, such as shoulder dystocia, bone fractures and nerve palsies. This study aimed to compare the plasma glucose concentrations and serum insulin, leptin and adiponectin in cord blood of babies of women (a) without gestational diabetes mellitus (GDM), (b) with mild GDM under routine care, or (c) mild GDM with treatment.

**Methods:**

95 women with mild GDM on oral glucose tolerance testing (OGTT) at one tertiary level maternity hospital who had been recruited to the ACHOIS trial at one of the collaborating hospitals and randomised to either Treatment (n = 46) or Routine Care (n = 49) and Control women with a normal OGTT (n = 133) were included in the study. Women with mild GDM (treatment or routine care group) and OGTT normal women received routine pregnancy care. In addition, women with treated mild GDM received dietary advice, blood glucose monitoring and insulin if necessary.

The primary outcome measures were cord blood concentrations of glucose, insulin, adiponectin and leptin.

**Results:**

Cord plasma glucose was higher in women receiving routine care compared with control, but was normalized by treatment for mild GDM (p = 0.01). Cord serum insulin and insulin to glucose ratio were similar between the three groups. Leptin concentration in cord serum was lower in GDM treated women compared with routine care (p = 0.02) and not different to control (p = 0.11). Adiponectin was lower in both mild GDM groups compared with control (Treatment p = 0.02 and Routine Care p = 0.07), while the adiponectin to leptin ratio was lower for women receiving routine care compared with treatment (p = 0.08) and control (p = 0.05).

**Conclusion:**

Treatment of women with mild GDM using diet, blood glucose monitoring and insulin if necessary, influences the altered fetal adipoinsular axis characteristic of mild GDM in pregnancy.

## Background

Mild gestational diabetes is a common complication of pregnancy, affecting up to 9% of pregnant women [1]. Significant maternal, fetal and neonatal morbidities are associated with disturbances of glucose homeostasis in pregnancy. Gestational diabetes mellitus (GDM) increases the risk of macrosomia and associated birth injuries, such as shoulder dystocia, bone fractures and nerve palsies. Treatment of mild GDM is known to reduce these adverse perinatal outcomes [2]. Longer-term adverse health effects on offspring born to mothers with GDM include obesity [3] and impaired intellectual development [4].

GDM and diabetes mellitus result from insulin resistance and inadequate compensatory insulin secretion. Together these reduce insulin stimulation of glucose uptake by skeletal muscle, reduce inhibition of glucose release by liver, and reduce insulin suppression of lipolysis and amino acid turnover [5]. In GDM, these disturbances in insulin action increase the concentrations of glucose and other nutrients such as lipids and amino acids in maternal blood, and also increase their transfer to the fetus [5]. This combined with increased production of anabolic hormones and growth factors, including insulin within the fetus, promote fetal growth, resulting in macrosomia and birth complications. These metabolic changes and endocrine adaptations in the fetus may also contribute to longer-term complications such as obesity.

Maternal GDM, maternal obesity or being large or small at birth predicts an increased risk of glucose intolerance or diabetes in childhood, adolescence and adulthood [3,6,7]. This may be mediated in part by an increased propensity to obesity from infancy onwards [5,6]. Adipose tissue is an endocrine tissue, which secretes cytokines, such as leptin, and TNF-alpha, IL-6, which are insulin antagonistic, and adiponectin, which is insulin sensitizing [8]. The adipoinsular axis regulates growth, appetite, energy expenditure, body composition and metabolism postnatally [8]. Increased adiposity increases plasma concentrations of leptin, and other adipocytokines [8], and decreases that of adiponectin [9-11]. Diabetes in pregnancy increases concentrations of leptin in cord blood, which correlate with increased fetal fat mass, but whether this occurs in GDM is not known [12-14]. Adiponectin is also present in cord blood, [15,16], but the impact of GDM treatment on adiponectin in cord blood and the effect of treatment of GDM on adipocytokines on both leptin and adiponectin are also unknown.

The study reported here was therefore undertaken to characterise the effects of mild GDM and treatment with diet, blood glucose monitoring, and insulin therapy if required on markers of adiposity and the adipoinsular axis in the fetus. The latter reflect the intrauterine environment and the fetal response to this and are possible predictors of longer-term outcomes, such as risk of obesity, insulin resistance and diabetes in childhood and adolescence [6].

It was hypothesized that maternal GDM increases cord blood concentrations of glucose, insulin and leptin, but decreases concentrations of adiponectin; and that treatment of GDM with maternal dietary advice, blood glucose monitoring and insulin therapy if required, would partially prevent these consequences for the newborn baby.

The aims of this study were to measure cord blood plasma glucose, cord blood serum insulin, leptin and adiponectin concentrations, and to determine the insulin to glucose and adiponectin to leptin ratios in cord blood of babies of three groups of pregnant women at one of the collaborating hospitals for the Australian Carbohydrate Intolerance Study in Pregnant Women (ACHOIS) trial; women without GDM (Control); women with mild GDM women receiving routine obstetric care (Routine Care), and women with mild GDM receiving treatment of dietary advice, blood glucose monitoring and insulin therapy if required (Treatment).

## Methods

Cord blood was collected from women randomised to the ACHOIS trial at one of the collaborating centres, the Women's and Children's Hospital (WCH), Adelaide, Australia. Women in the ACHOIS trial had mild gestational diabetes mellitis on oral glucose tolerance test (defined by a 75 g OGTT fasting venous plasma glucose <7.8 mmol/1 and/or 2 hour ≥7.8 mmol/L and ≤ 11.0 mmol/1 between 24 – 34^6 ^weeks gestation [17]). Ninety-five of the 157 women randomised to the ACHOIS trial at the WCH gave written informed consent to participate in this study and cord blood samples were able to be collected during the study period. Forty-six women had been randomised to the Treatment Group and received dietary advice, blood glucose monitoring and insulin as necessary as recommended by clinical practice guidelines [18], and 49 women had been randomised to the Routine Care group and received routine pregnancy care. At the WCH glucose concentrations are not routinely monitored during labour in women with GDM and no glucose infusion is routinely given. Details of the methodology used in the ACHOIS trial have been reported previously [2]. A Control group of 133 women with a normal OGTT was also recruited from the WCH during the time course of this study and gave written informed consent to participate. The Control group received routine pregnancy care. Approval for the study was given by the Institutional Ethics Committee.

Cord blood samples were collected at birth by delivery suite staff. The cord was milked to produce a sample of arterial and venous blood, and was placed in tubes containing sodium fluoride for glucose analysis, and tubes containing clot retraction accelerator for serum analysis of insulin, leptin and adiponectin. Samples were centrifuged after collection at 5000 rpm for 10 minutes at 4°C.

Samples were assayed blind. Plasma glucose concentrations were measured in duplicate using the Beckman SYNCHRON CX Systems assay using hexokinase and were accurate in the range of 0.3–38.8 mmol/l. Serum insulin, leptin and adiponectin concentrations were measured using commercially available radioimmunoassay kits specific for the human hormones, obtained from Linco Research Pty Ltd. Iodine-125 activity was measured on the Wallac 1261 Multigamma Gamma Counter.

### Power calculation

In order to detect a difference in serum adiponectin concentrations of 4.3 ng/mL between the two GDM groups and the Control group, and of 5.2 ng/mL between the two GDM groups, a sample size of 47 women for each of the GDM groups and 132 women in the Control group was required, with an estimated standard deviation of 8.9, using a two-sided significance level of 5% and power of 80%.

### Statistical analysis

Statistical analysis of glucose and hormone parameters was carried out using SAS Software (SAS Institute Inc., USA). Data that were not normally distributed were log-transformed, and were used for statistical analysis to approximate normal distributions. Analysis of variance was used to compare outcomes among treatment groups. Initial analyses were unadjusted. Adjustments were then made where appropriate for parity, gestational age at birth, fetal sex, smoking, birth weight and maternal body mass index. Statistical significance was defined as p < 0.05.

## Results

The maternal characteristics at entry to the study in the different study groups were similar (Table 1). Four women in the Treatment group received insulin therapy in the antenatal period and one woman in the Routine Care group. There were no differences in mode of birth between the groups.

### Cord blood plasma glucose

Plasma glucose was increased by about 10% in babies in the Routine Care group, compared with those in the Control group (p = 0.01) (Table 2). No difference was detected in plasma glucose between babies in the Treatment and Control groups, or between babies in the Treatment and Routine Care groups. However, after adjustment for parity, smoking, gestational age at birth and fetal sex, babies in both GDM (Treatment and Routine Care) groups had increased plasma glucose (+~10%) compared with babies in the Control group (Table 3).

### Cord blood serum insulin and serum insulin/plasma glucose ratio (Tables 2 &3)

No differences were detected between the three study groups in either cord blood serum insulin or serum insulin/glucose ratios in unadjusted or adjusted analyses (adjusted for parity, smoking, gestational age at birth, fetal sex, birth weight or maternal body mass index).

### Cord blood serum adiponectin

No difference was detected in serum adiponectin between babies in the Routine Care group and the Control group (p = 0.07) (Table 2). Babies of women in the Treatment group had around 16% lower serum adiponectin compared with the Control group (p = 0.02). No difference was detected between serum adiponectin in babies of women in the Treatment group and the Routine Care group.

After adjustment for parity, smoking, gestational age at birth, fetal sex and maternal body mass index, serum adiponectin was around 15% lower in the Routine Care group (p = 0.04), and around 23% lower in the Treatment group (p = 0.01) than in the Control group. Serum adiponectin was similar in both GDM groups. Further adjustment for birth weight did not alter these outcomes (Table 3).

### Cord blood serum leptin

Leptin concentrations were around 22% higher in cord blood in the Routine Care group compared with the Treatment group (p = 0.02) (Table 2). This difference persisted after adjustment for parity, smoking, gestational age at birth and sex. However, when adjustment was also made for maternal body mass and birth weight no difference between the two GDM groups could be detected. No difference in serum leptin was detected between babies in the either of the GDM groups compared with the Control group in unadjusted and adjusted analyses (adjusted for parity, smoking, gestational age at birth, sex, maternal body mass and birth weight) (Table 3).

### Cord blood serum adiponectin/leptin ratio

The adiponectin/leptin ratio was around 38% lower in the Routine Care group compared with the Control group (p = 0.05), even when adjusted for parity, smoking, gestational age at birth and sex. (Table 2). However, this difference disappeared after adjustment for birth weight (p = 0.25) (Table 3). No difference was detected between the adiponectin/leptin ratios in the Treatment group and the Control group, or between the Treatment and Routine Care groups (p = 0.08) in adjusted or unadjusted analyses (adjustment for parity, smoking, gestational age at birth and sex and birth weight). Adjustment for maternal body mass index had no effect on any of these outcomes.

## Discussion

Mild maternal GDM alters cord blood concentrations of glucose and adiponectin in the neonate, and these changes can be partly reversed by treatment with dietary advice, blood glucose monitoring and insulin therapy as necessary. Babies of women with mild gestational diabetes in the Routine Care group exhibited hyperglycaemia and endocrine markers of increased adiposity, which is characteristic of infants of diabetic mothers [5].

While babies of women with mild GDM in pregnancy receiving routine care, had increased cord blood glucose concentrations, the concentration of insulin in cord serum and the insulin to glucose ratios were unchanged. This suggests that the fetus experienced hyperglycaemia, but did not respond with significant hyperinsulinaemia. These findings are at odds with Roach *et al *[19], where cord blood insulin was increased, however the current study is substantially larger. Furthermore, babies of women with mild GDM in the Routine Care group had increased concentrations of leptin in cord serum compared with babies of women in the Treatment group. consistent with those reported for babies of mothers with GDM [20] or with diabetes [12,14,21].

Leptin in cord blood is derived mainly from fetal adipose tissue, although the placenta may contribute, and correlates with birth weight and adipose tissue mass [22-24]. The increased cord blood leptin reported here with mild GDM in the Routine Care group, therefore suggests increased fetal fat deposition has occurred and may contribute to the fetal macrosomia and dystocia, as seen in GDM [13]. However, infant fat mass was not directly assessed in the current study. Leptin acts centrally at the hypothalamus to regulate appetite and to increase energy expenditure [25]. If the hyperleptinaemia of the baby born to women with untreated mild GDM is due substantially to increased adiposity, it may persist for some time. This could reduce appetite and food intake and might contribute to the phenomenon of catch-down growth seen in macrosomic infants following birth [26].

Babies of women with mild GDM in both Treatment and the Routine Care group exhibited reduced cord blood concentrations of adiponectin compared with those in the Control Group, when the analysis was adjusted for parity, smoking, gestational age at birth, fetal sex, birth weight, and maternal body mass, as previously described in babies born to diabetic women [27]. This has not been examined previously in GDM. Increased adiposity in these babies reduces circulating adiponectin as in the adult [10,15], possibly as a result of increasing adipocyte size, maturation and aging [28,29]. Stress-related hormones and cytokines, such as glucocorticoids, catecholamines and TNF-α can inhibit adiponectin production however [30], and could be increased if these babies have a higher metabolic rate and oxygen deficit chronically in utero or during labor.

Similarly, the adiponectin to leptin ratio in cord blood was reduced in babies of mothers with mild GDM in the Routine Care Group further supporting a shift in the balance of adipocytokines to a profile which is more antagonistic of insulin action. While cord blood leptin levels are increased in the cord blood of babies of diabetic women [12] and women with GDM [31], the concentrations of adiponectin and their ratio with leptin in cord blood have not been characterized to date. This study has therefore demonstrated that mild gestational diabetes in women receiving routine care exposes the fetus to hyperglycaemia, but not significant hyperinsulinaemia, but nevertheless, results in markers of increased adiposity, hyperleptinaemia and reduced absolute and relative adiponectin levels. This cytokine profile resembles that of infants of diabetic mothers [12,14,21,27,32] and is one that might impair insulin action in these neonates, altering subsequent growth and metabolic control.

The range of concentrations of adiponectin in cord serum reported here are comparable to those reported in other studies of neonates from normal or diabetes pregnancies [15,16] and similarly, are much higher than in adults [33]. Recent studies have shown that adiponectin circulates as hexamers and large molecular weight multimers, which appear to target different tissues in affecting insulin sensitivity and metabolism [34]. The RIA used in the current study measures total adiponectin and the impact of mild GDM and treatment on various forms of adiponectin may give more insight into the likely impact of any changes on fetal and infant growth and metabolism. Because other cytokines, IL-6 and TNF-alpha are strongly implicated in adipocyte regulation of insulin action, it would be of interest to examine these in the same cohorts to help identify mediators contributing to the outcomes for the neonatal adipoinsular axis.

This study has shown that treatment of women with mild GDM did not lower cord blood glucose to that of women without mild GDM. Treatment of GDM therefore does not reduce exposure of the fetus to hyperglycaemia. However, treatment of women with mild GDM did lower concentrations of leptin in cord serum. This suggests that some of the other potentially adverse consequences of mild GDM for body composition may have been prevented by treatment.

Adaptations to an altered environment during intrauterine development such as that induced by gestational diabetes during pregnancy, may lead to permanent changes in the makeup of the human body [35]. Previous studies [3,6,36] have shown that infants of women with GDM have an increased risk of adolescent obesity and glucose intolerance. A recent study reported that fasting plasma adiponectin concentrations in adults could predict subsequent changes in insulin sensitivity over several years [37]. A recent study reported lower concentrations of adiponectin in young adults who had been born small for gestational age [38]. Since low and high birth weight are factors predisposing to gestational diabetes and type 2 diabetes in adult life [39], it will be important to determine if the partially normalized adiponectin-leptin ratio in cord blood is associated with improved insulin sensitivity or glucose tolerance later in life in these children. The decreased cord blood adiponectin may be predictive of insulin resistance in infancy and possibly childhood, increasing the risk of developing related disorders.

## Conclusion

In summary, babies of women with untreated mild GDM compared with women without GDM have increased glucose and reduced adiponectin, but unaltered insulin levels and insulin to glucose ratio in cord blood. Treatment of mild GDM with dietary advice, blood glucose monitoring and insulin as necessary, did not lower cord blood glucose or increase adiponectin, but did reduce cord blood leptin and increase the adiponectin to leptin ratio.

## Competing interests

The author(s) declare that they have no competing interests.

## Authors' contributions

Louise Pirc carried out the assays and prepared the initial draft of the manuscript. Julie Owens participated in the design of the study, performed the statistical analyses and contributed to writing of all drafts of the manuscript. Caroline Crowther designed the study, co-ordinated collection of samples, assisted in interpretation of the data and drafting the manuscript. Kristyn Willson performed the data analyses, Miles De Blasio performed the assays and commented on all drafts of the manuscript. Jeffrey Robinson contributed to study design, interpretation of the data and preparation of all drafts of the manuscript. All authors read and approved the final manuscript.

## Pre-publication history

The pre-publication history for this paper can be accessed here:



## Figures and Tables

**Table 1:** Maternal Characteristics at Study Entry

	**Mild GDM**				**Not GDM**	
	
	**Treatment Group**		**Routine Care Group**		**Control Group**	
	
**Characteristic**	**n = 46**		**n = 49**		**n = 133**	
**Maternal Age (years)**	31.7	5.2	30.1	4.4	29.2	5.5
Parity*						
0	25	54.3	32	65.3	74	55.6
1–3	20	43.5	16	32.7	55	41.4
4 +	1	2.2	1	2.0	4	3.0
**BMI (kg/m**^2^**)**^†^	26	23.9–32.0	25.8	22.8–29.4	24.9	22.4–29.1
Smoking*	6	13.0	5	10.2	26	19.5

Figures are mean and standard deviation, number and percentages*, or median and interquartile range^†^.

**Table 2:** Cord/Plasma/Serum concentrations of glucose and cytokines with adjustments for parity, smoking, gestational age at birth and fetal sex

	**GDM**								**Not GDM**			
	
	**Treatment Group**				**Routine Care Group**				**Control Group**			
	
	Unadjusted		Adjusted*		Unadjusted		Adjusted*		Unadjusted		Adjusted*	
**Plasma Glucose (mmol/ml)**	4.8	0.2	4.7	0.2^+^	5.0	0.2^+^	4.8	0.2^+^	4.5	0.1	4.3	0.2
**Serum Insulin (μU/ml)^**	2.0	0.1	2.0	0.2	2.2	0.1	2.2	0.2	2.1	0.1	2.0	0.1
**Insulin to Glucose Ratio^**	0.5	0.1	0.5	0.2	0.6	0.1	0.6	0.2	0.6	0.1	0.5	0.1
**Serum Adiponectin (ng/ml)**	23.0	1.7^+^	21.8	2.1^+^	24.3	1.5	23.2	2.0^+^	27.5	1.0	26.8	1.7
**Serum Leptin (ng/ml)^**	2.2	0.1	2.2	0.2	2.7	0.1^#^	2.6	0.1^#^	2.4	0.1	2.4	0.1
**Adiponectin to Leptin Ratio^**	0.8	0.2	0.8	0.2	0.5	0.1^+^	0.5	0.2^+^	0.8	0.1	0.8	0.1

Figures are mean, standard error

^ Statistical analysis was performed on log-transformed data

* Results adjusted for parity, smoking, gestational age at birth and fetal sex

^# ^p ≤ 0.05 versus GDM (Treatment Group)

^+ ^p ≤ 0.05 versus Control Group

**Table 3:** Cord Plasma/Serum concentrations of glucose and cytokines with additional adjustments for birth weight and maternal body mass index

	**GDM**								**Not GDM**			
	
	**Treatment Group**				**Routine Care Group**				**Control Group**			
	
	BW*		BW+BMI*		BW*		BW+BMI*		BW*		BW+BMI*	
**Plasma Glucose (mmol/ml)**	4.7	0.2	4.7	0.2	4.8	0.2^+^	4.9	0.2^+^	4.3	0.2	4.3	0.2
**Serum Insulin (μU/ml)^**	2.0	0.2	2.1	0.2	2.1	0.2	2.1	0.2	2.0	0.1	2.0	0.1
**Insulin to Glucose Ratio^**	0.6	0.2	0.6	0.2	0.6	0.2	0.5	0.2	0.6	0.1	0.5	0.1
**Serum Adiponectin (ng/ml)**	22.1	2.2^+^	21.4	2.2^+^	23.0	2.0^+^	23.6	2.1^+^	27.0	1.7	27.8	1.8
**Serum Leptin (ng/ml)^**	2.4	0.1	2.4	0.1	2.5	0.1	2.5	0.1	2.4	0.1	2.5	0.1
**Adiponectin to Leptin Ratio^**	0.7	0.2	0.6	0.2	0.6	0.2	0.6	0.2	0.8	0.1	0.8	0.1

Figures are mean, standard error

BW = Birth weight

BW + BMI = Birth weight + Maternal BMI

* Results also adjusted for parity, smoking, gestational age at birth and sex, with additional adjustment for BW and BMI

^ Statistical analysis was performed on log-transformed data

^# ^p ≤ 0.05 versus GDM (Routine Care Group)

^+ ^p ≤ 0.05 versus Control Group
